# hopsy — a methods marketplace for convex polytope sampling in Python

**DOI:** 10.1093/bioinformatics/btae430

**Published:** 2024-07-01

**Authors:** Richard D Paul, Johann F Jadebeck, Anton Stratmann, Wolfgang Wiechert, Katharina Nöh

**Affiliations:** Institute of Bio- and Geosciences, IBG-1: Biotechnology, Forschungszentrum Jülich, 52428 Jülich, Germany; Institute of Advanced Simulations, IAS-8: Data Analytics and Machine Learning, Forschungszentrum Jülich, 52428 Jülich, Germany; Institute of Bio- and Geosciences, IBG-1: Biotechnology, Forschungszentrum Jülich, 52428 Jülich, Germany; Computational Systems Biotechnology (AVT.CSB), RWTH Aachen University, 52074 Aachen, Germany; Institute of Bio- and Geosciences, IBG-1: Biotechnology, Forschungszentrum Jülich, 52428 Jülich, Germany; Computational Systems Biotechnology (AVT.CSB), RWTH Aachen University, 52074 Aachen, Germany; Institute of Bio- and Geosciences, IBG-1: Biotechnology, Forschungszentrum Jülich, 52428 Jülich, Germany; Computational Systems Biotechnology (AVT.CSB), RWTH Aachen University, 52074 Aachen, Germany; Institute of Bio- and Geosciences, IBG-1: Biotechnology, Forschungszentrum Jülich, 52428 Jülich, Germany

## Abstract

**Summary:**

Effective collaboration between developers of Bayesian inference methods and users is key to advance our quantitative understanding of biosystems. We here present hopsy, a versatile open-source platform designed to provide convenient access to powerful Markov chain Monte Carlo sampling algorithms tailored to models defined on convex polytopes (CP). Based on the high-performance C++ sampling library HOPS, hopsy inherits its strengths and extends its functionalities with the accessibility of the Python programming language. A versatile plugin-mechanism enables seamless integration with domain-specific models, providing method developers with a framework for testing, benchmarking, and distributing CP samplers to approach real-world inference tasks. We showcase hopsy by solving common and newly composed domain-specific sampling problems, highlighting important design choices. By likening hopsy to a marketplace, we emphasize its role in bringing together users and developers, where users get access to state-of-the-art methods, and developers contribute their own innovative solutions for challenging domain-specific inference problems.

**Availability and implementation:**

Sources, documentation and a continuously updated list of sampling algorithms are available at https://jugit.fz-juelich.de/IBG-1/ModSim/hopsy, with Linux, Windows and MacOS binaries at https://pypi.org/project/hopsy/.

## 1 Introduction

Models are central to systems biology, acting as gateways to generate insights, making predictions, or testing hypotheses. The types of models used are diverse, ranging from statistical to physics-based. For operating models as epistemological tools, two steps are essential: exploration of the models’ capacities to represent data and estimation of model parameters from data. For both, recent years have witnessed a surge of interest in Bayesian statistics, expressing the desired information in the form of probability density functions (PDFs), under the notion of uncertainty (Wilkinson 2006).

In many cases, the model definition spaces are (explicitly or implicitly) bounded by linear half-spaces making up a convex polytope (CP), for reasons as diverse as physiological limitations, energetic or other resource constraints, or mass balances operated at steady-state (Liebermeister and Noor 2021). Premier examples are (bio)chemical reaction networks (Heinken *et al.* 2023), or ecosystem models (Gellner *et al.* 2023). Actually, CP-constrained models also appear in a wide range of domains outside biology, such as gravitational lensing (Lubini *et al.* 2013), smart power grids (Theorell and Stelling 2022), or transport planning (Airoldi and Blocker 2013).

CP-constrained PDFs, defined by real-world models, are high-dimensional and rarely analytically tractable. To approximate the PDFs, Markov chain Monte Carlo (MCMC) is a well-established sampling approach, with numerous variants implemented in powerful probabilistic programming tools (Carpenter *et al.* 2017, Abril-Pla *et al.* 2023). However, such general MCMC algorithms fail to solve CP-constrained sampling problems efficiently because neglecting the CP geometry results in high rejection rates or ineffective space exploration (Jadebeck *et al.* 2023, Supplementary Section S2).

Kannan *et al.* 1997, Kannan and Narayanan 2012). Here, propelled by the needs in the metabolic modeling domain, various open packages have become available (Heirendt *et al.* 2019, Chalkis and Fisikopoulos 2021, Ciomek and Kadziński 2021, Jadebeck *et al.* 2021). This commoditization of uniform CP-constrained sampling, along with theoretical advances (Laddha and Vempala 2021), has empowered metabolic researchers to approach increasingly high-dimensional problems within the domain (Thiele *et al.* 2020, Jadebeck *et al.* 2023), and beyond (Gellner *et al.* 2023).

For nonuniform CP sampling the situation is, however, quite different. Here, despite much work on “standardized” Gaussian and log-concave PDFs exists (Kook *et al.* 2022, Chalkis, Fisikopoulos *et al.* 2023), the application as well as algorithmic landscapes for CP-constrained PDF sampling are scattered. Stimulated by the successes in uniform CP sampling, sampling of general nonuniform CP-constrained PDFs should be equally simple and accessible for domain experts. In turn, such a solution empowers MCMC developers to benchmark, improve and create new algorithms using real-world applications posed by domain experts.

Borrowing from the idea of a marketplace, we present the open-source Python package hopsy. hopsy is a flexible platform for general CP-constrained PDF sampling that seamlessly connects domain-specific simulation software and modern MCMC algorithms, via minimal and expressive interfaces. Specifically, hopsy leverages Python to allow domain experts and MCMC researchers to quickly implement and share domain-specific MCMC sampling workflows, while offering high-performance state-of-the-art implementations and support for common and innovative applications.

## 2 Approach and implementation

By design, hopsy is a “batteries-included” platform to support convenient MCMC sampling of general CP-constrained PDFs, independent of the application domain. To facilitate flexibility at a low entry barrier, hopsy is implemented in Python and takes advantage of the C++-library for highly optimized polytope sampling HOPS (Jadebeck *et al.* 2021). Performance critical code from HOPS is integrated via pybind11 (https://github.com/pybind/pybind11) while convenience functions are implemented in Python.

**Figure 1.**
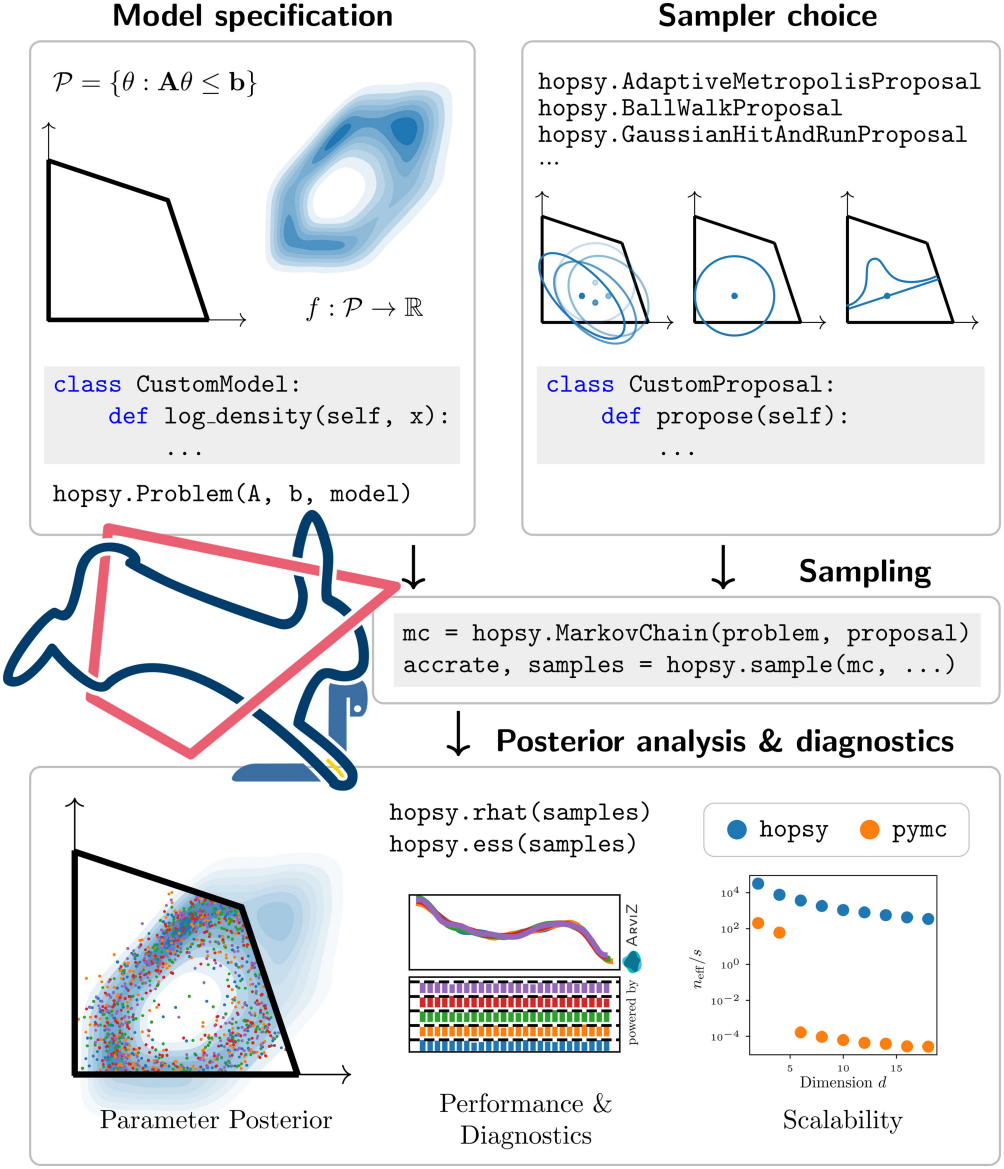
Convex polytope-constrained sampling workflow with hopsy.

In the hopsy sampling workflow (Fig. 1), a model is specified by defining the PDF on a CP-constrained support. Then a suitable sampling algorithm is selected, configured and run. For a continuously updated listing of MCMC algorithms, we refer to https://modsim.github.io/hopsy/userguide/sampling.html#proposals. After the sampling step, convergence diagnostics and visualizations are provided by the widely used ArviZ package (Kumar *et al.* 2019).

The model specification consists of an explicit CP formulation in half-space representation and a PDF (by convention expected as log-density):

(1) $$P=\{\theta \in R^{n}|A\cdot \;\theta \leq b\},\;f:P\to R,\;\theta \mapsto f(\theta )$$

with unknown model parameters $\theta \in R^{n},\;A\in R^{n\times m}$ and $b\in R^{m}$. To obtain an explicit CP formulation from an implicit one, e.g. an underdetermined linear equation system, linear algebra recipes exist (Supplementary Section S3). The log-density *f* may be given as the logarithm of a closed-form PDF or, in the case of Bayesian inference, as a log-posterior provided by a simulation code. Note that the model specification in Eq. (1) is well-posed only if the resulting density function is integrable. In cases where the gradient or the curvature of the log-density *f* is available, these can be utilized for proposal construction, e.g. for Riemannian-type MCMC algorithms (Gatmiry and Vempala 2022). Ready-to-use model specifications for standard polytopes (simplices, Birkhoff polytopes), and common log-densities are also available (see documentation at https://modsim.github.io/hopsy/).

The central entity that encapsulates the model specification Eq. (1) is the hopsy-class hopsy. Problem. The CP is passed in the form of domain-agnostic NumPy arrays (Harris *et al.* 2020) and the log-density is a Python object with a log_density-method, as well as optionally, log_gradient and/or positive-definite log_curvature methods. hopsy’s open plugin-architecture for custom Python code makes the formulation of the model ingredients extremely flexible. Thereby, models are either directly specified in Python or by calling external ODE- or PDE-solvers, facilitating the construction of composite models. The high flexibility for posing the log-density is achieved by a combination of interface classes and a *trampoline* (https://github.com/pybind/pybind11), which redirects calls from HOPS to custom log-densities defined in Python. Using the same trampoline-technique, hopsy supports custom proposal implementations in Python, which seamlessly integrate with model specifications. Similarly to the hopsy. Model, a proposal in hopsy needs to implement a minimal interface consisting of a constructor expecting at least a hopsy. Model, an initial state, a propose method, which creates a new proposal based on the current state, and, for nonsymmetric proposal distributions, the log_acceptance_probability method to obtain the correct acceptance probability in the Metropolis filter. Importantly, this eliminates the need to write tedious “glue-code” to connect existing implementations for Eq. (1) to newly designed proposals. Examples of how easy it is to integrate and combine custom PDFs and tailor-made MCMC algorithms are given in the next section.

The model specification and user-selected sampling algorithm are collected in the hopsy. MarkovChain class. The function hopsy.sample advances a set of Markov chains by the number of requested samples. hopsy generates random numbers using statistically reliable 64 bit permuted congruential generators (O’Neill 2014) with 128 bit states and periods of 2^128^, which is sufficient to prevent repeat of random numbers in realistic settings. Because instances of hopsy. MarkovChain support the pickle functionality of Python, check-pointing sampling runs is simple. The hopsy.sample function stores samples either in-memory as NumPy-arrays or in (remote) databases (https://github.com/pymc-devs/mcbackend).

Further highlight features of hopsy are CP pre-processing by rounding using PolyRound (Theorell *et al.* 2021), feasibility checks for CPs, MCMC acceptance-rate tuning (Roberts and Rosenthal 2001), parallel tempering (PT) (Geyer 1991, Hukushima and Nemoto 1995) for tackling multi-modal log-densities, and Reversible Jump MCMC (Green 1995, Theorell and Nöh 2019). Software quality is ensured by a continuous integration (CI) pipeline for automatic testing, automatic code-style checks using pre-commit, changelogs, documentation including example Jupyter notebooks, and semantic versioning.

## 3 Showcasing hopsy

To demonstrate the flexibility of hopsy, we selected the field of metabolic modeling, which stands out due to its high-dimensional inference tasks within a plethora of different inference approaches.

Uniform CP sampling is a common approach for unbiased exploration of metabolic capabilities (Haraldsdóttir *et al.* 2017, Supplementary Section S3). Compared to the state-of-the-art Coordinate Hit-and-Run with Rounding and Thinning (CHRRT) algorithm implemented in HOPS (Jadebeck *et al.* 2023), we ponder on alternate proposals, specifically Over-relaxed Hit-and-Run (de Concini and de Martino 2015), and a recent adaptive sampling approach that uses iterative singular value decomposition (SVD) transformations of the parameter space instead of polytope rounding (Chalkis *et al.* 2023). Exploiting the flexibility of the open plugin-architecture, implementing the alternate proposals took only few lines of Python code (30 for Over-relaxed Hit-and-Run and 49 for iterative SVD-based rounding). We benchmarked the three MCMC algorithms for a synthetic problem (16D Birkhoff polytope), and two *Escherichia coli* models. For the synthetic problem and one of the *E. coli* models the adaptive sampling approach were the most efficient, while for the other *E. coli* model CHRRT was found best (Supplementary Fig. S3). The result underlines the intricacy of CP sampling and, thus, the importance of proposal design and testing, even for similar models of the same organism. hopsy’s performance is competitive with packages specialized for uniform sampling on commodity computers (Chalkis *et al.* 2024), while also benefiting from supercomputer hardware for high-dimensional problems (Supplementary Fig. S5).

A key quantity in metabolic models are reaction rates (fluxes) that are inferred from isotopic labeling data, in a framework known as Bayesian ^13^C-MFA (Theorell *et al.* 2017) (Supplementary Section S4). Here, the simulation of ^13^C-labeled metabolites entertains a nonlinear mapping from the flux parameter space to the observation space. consequently, the sum-of-squares residual between simulated and measured ^13^C-labeled metabolites gives rise to a nonuniform CP-constrained PDF. Since efficient simulation is key, the ^13^C-model was called via the domain-specific high-performance simulator 13CFLUX2 (Weitzel *et al.* 2012). The Hit-and-Run with Rounding algorithm was used without and with PT for sampling one of the above-mentioned *E. coli* models. Our results show that (i) PT led to improved mixing, as visible in the trace plots and Gelman-Rubin diagnostic (Supplementary Fig. S6), and (ii) compared to uniform sampling, incorporating ^13^C data strongly reduced the flux parameter uncertainty (Supplementary Fig. S7), and predict likely measurement distributions for isotope labeling experiments (Supplementary Fig. S8).

In a third application, we highlight the flexibility of hopsy by creating a composite model using the trampoline (Supplementary Section S5). Conventionally, extracellular rates are first estimated by means of so-called bioprocess models. Once estimated, they are incorporated into the ^13^C-model as external rate measurements together with their (symmetrized) standard deviations. From a statistical standpoint, simultaneous estimation of bioprocess and ^13^C-model parameters promises a better understanding of parameter correlations. Therefore, in a rapid prototyping manner, we built a new composite model with which bioprocess and flux parameters are estimated simultaneously. Comparing the outcome with the conventional modeling procedure in Supplementary Fig. S9 indeed shows an information gain. Thus, with hopsy it was simple to quickly test a new promising modeling idea in the field of ^13^C-MFA.

## 4 Conclusion

hopsy is a mature open-source Python toolbox for highly optimized polytope sampling. hopsy strips the polytope sampling problem down to its minimal formal requirements and provides a simple, extensible interface. This makes hopsy applicable to a broad range of polytope sampling problems, such as exploring polytopic parameter spaces by uniform sampling, testing MCMC approaches, and efficiently tackling challenging Bayesian inference problems. Furthermore, we demonstrate that it is easy with hopsy to implement novel (composite) modeling approaches. Our showcases, despite being from the field of metabolic modeling, are inspirations for other scientific fields, such as ecological modeling (Gellner *et al.* 2023), optimization of chromatography pipelines (Schmölder and Kaspereit 2020), or single-cell analysis (Paul *et al.* 2024), unlocking a broad portfolio of applications for method developers. hopsy thus fertilizes collaboration between domain experts and MCMC developers by facilitating easy sharing of new problems and MCMC approaches, allowing both communities to bring their fruits into practice quicker.

## Supplementary Material

btae430_Supplementary_Data

## Data Availability

The data underlying this article is publicly available in the provided JuGit repository.

## References

[btae430-B30] Abril-Pla O, Andreani V, Carroll C et al PyMC: a modern and comprehensive probabilistic programming framework in Python. PeerJ Comput Sci 2023;9:e1516. 10.7717/peerj-cs.1516 PMC10495961 37705656

[btae430-B1] Airoldi EM , Blocker AW. Estimating latent processes on a network from indirect measurements. JASA 2013;108:149–64.

[btae430-B2] Bélisle CJP , Romeijn HE, Smith RL. Hit-and-Run algorithms for generating multivariate distributions. Math OR 1993;18:255–66.

[btae430-B3] Carpenter B , Gelman A, Hoffman MD et al Stan: a probabilistic programming language. J Stat Softw 2017;76:1–32. 36568334 10.18637/jss.v076.i01 PMC9788645

[btae430-B4] Chalkis A, Emiris I, Fisikopoulos V et al Geometric algorithms for sampling the flux space of metabolic networks. J Comput Geom 2023; 14:195–220.

[btae430-B5] Chalkis A , Fisikopoulos V. VolEsti: volume approximation and sampling for convex polytopes in R. R J 2021;13:642–60.

[btae430-B6] Chalkis A , Fisikopoulos V, Papachristou M et al Truncated log-concave sampling for convex bodies with reflective Hamiltonian Monte Carlo. ACM Trans Math Softw 2023;49:1–25.

[btae430-B7] Chalkis A , Fisikopoulos V, Tsigaridas E et al dingo: a Python package for metabolic flux sampling. Bioinform Adv 2024;4:vbae037. 38586119 10.1093/bioadv/vbae037 PMC10997433

[btae430-B8] Ciomek K , Kadziński M. Polyrun: a java library for sampling from the bounded convex polytopes. SoftwareX 2021;13:100659.

[btae430-B9] de Concini G , de Martino D. Over-relaxed hit-and-run Monte Carlo for the uniform sampling of convex bodies with applications in metabolic network biophysics. Int J Mod Phys C 2015;26:1550010.

[btae430-B10] Gatmiry K , Vempala SS. Convergence of the Riemannian Langevin algorithm. arXiv, arXiv:2204.10818, 2022, preprint: not peer reviewed.

[btae430-B11] Gellner G , McCann K, Hastings A. Stable diverse food webs become more common when interactions are more biologically constrained. Proc Natl Acad Sci USA 2023;120:2017. 10.1073/pnas.2212061120 PMC10400988 37487080

[btae430-B13] Geyer CJ. Markov chain Monte Carlo maximum likelihood. In: Keramidas EM (ed.) *Computing Science and Statistics: Proceedings of the 23rd Symposium on the Interface*, Fairfax Station, VA, USA: Interface Foundation of North America, 1991, 156–163.

[btae430-B14] Green PJ. Reversible jump Markov chain Monte Carlo computation and Bayesian model determination. Biometrika 1995;82:711–32.

[btae430-B15] Hukushima K , Nemoto K. Exchange Monte Carlo method and application to spin glass simulations. J Phys Soc Jpn 1995;65:1604–8.

[btae430-B16] Haraldsdóttir HS , Cousins B, Thiele I et al CHRR: coordinate hit-and-run with rounding for uniform sampling of constraint-based models. Bioinformatics 2017;33:1741–3. 28158334 10.1093/bioinformatics/btx052 PMC5447232

[btae430-B17] Harris CR , Millman KJ, van der Walt SJ et al Array programming with NumPy. Nature 2020;585:357–62. 32939066 10.1038/s41586-020-2649-2 PMC7759461

[btae430-B18] Heinken A , Hertel J, Acharya G et al Genome-scale metabolic reconstruction of 7,302 human microorganisms for personalized medicine. Nat Biotechnol 2023;41:1320–31. 36658342 10.1038/s41587-022-01628-0 PMC10497413

[btae430-B19] Heirendt L , Arreckx S, Pfau T et al Creation and analysis of biochemical constraint-based models using the COBRA toolbox v.3.0. Nat Protoc 2019;14:639–702. 30787451 10.1038/s41596-018-0098-2 PMC6635304

[btae430-B20] Jadebeck JF , Theorell A, Leweke S et al HOPS: high-performance library for (non-) uniform sampling of convex-constrained models. Bioinformatics 2021;37:1776–7. 33045081 10.1093/bioinformatics/btaa872

[btae430-B21] Jadebeck JF , Wiechert W, Nöh K. Practical sampling of constraint-based models: optimized thinning boosts CHRR performance. PLoS Comput Biol 2023;19:e1011378. 37566638 10.1371/journal.pcbi.1011378 PMC10446239

[btae430-B22] Kannan R , Narayanan H. Random walks on polytopes and an affine interior point method for linear programming. Math OR 2012;37:1–20.

[btae430-B23] Kannan R , Lovász L, Simonovits MS. Random walks and an O*(n^5^) volume algorithm for convex bodies. Random Struct Alg 1997;11:1–50.

[btae430-B24] Kook Y , Lee YT, Shen R et alSampling with Riemannian Hamiltonian Monte Carlo in a constrained space. In: Koyejo S, Mohamed S, Agarwal A et al (eds), Adv Neural Inf Process Syst, Vol. 35. Red Hook, NY, USA: Curran Associates, Inc., 2022, 31684–96.

[btae430-B25] Kumar R , Carroll C, Hartikainen A et al Arviz a unified library for exploratory analysis of Bayesian models in Python. JOSS 2019;4:1143.

[btae430-B26] Laddha A , Vempala SS. Convergence of Gibbs sampling: coordinate Hit-and-Run mixes fast. In: Buchin K, de Verdière ÉC (eds.), 37th Int. Symp. on Computational Geometry (SoCG 2021), Vol. 189 of Leibniz Int. Proc. Inform. (LIPIcs), Germany: Dagstuhl, 2021, 51:1–51.12.

[btae430-B27] Liebermeister W , Noor E. Model balancing: a search for in-vivo kinetic constants and consistent metabolic states. Metabolites 2021;11:749. 34822407 10.3390/metabo11110749 PMC8621975

[btae430-B28] Lubini M , Sereno M, Coles J et al Cosmological parameter determination in free-form strong gravitational lens modelling. MNRAS 2013;437:2461–70.

[btae430-B29] O’Neill ME. PCG: a family of simple fast space-efficient statistically good algorithms for random number generation. Technical Report HMC-CS-2014-0905, Harvey Mudd College, Claremont, CA, 2014.

[btae430-B31] Paul RD , Seiffarth J, Scharr H et al Robust approximate characterization of single-cell heterogeneity in microbial growth. In: *IEEE Proceedings of the 22th International Symposium on Biomedical Imaging (ISBI)*, Athens, Greece, 2024.

[btae430-B32] Roberts GO , Rosenthal JS. Optimal scaling for various Metropolis-Hastings algorithms. Stat Sci 2001;16:351–67.

[btae430-B33] Schmölder J , Kaspereit M. A modular framework for the modelling and optimization of advanced chromatographic processes. Processes 2020;8:65.

[btae430-B34] Theorell A , Nöh K. Reversible jump MCMC for multi-model inference in metabolic flux analysis. Bioinformatics 2019;36:232–40. 10.1093/bioinformatics/btz500 31214716

[btae430-B35] Theorell A , Stelling J. Metabolic networks, microbial consortia, and analogies to smart grids. Proc IEEE 2022;110:541–56.

[btae430-B36] Theorell A , Leweke S, Wiechert W et al To be certain about the uncertainty: Bayesian statistics for ^13^C metabolic flux analysis. Biotechnol Bioeng 2017;114:2668–84. 28695999 10.1002/bit.26379

[btae430-B37] Theorell A , Jadebeck JF, Nöh K et al Polyround: polytope rounding for random sampling in metabolic networks. Bioinformatics 2021;38:556–67. 10.1093/bioinformatics/btab552 PMC8723145 34329395

[btae430-B38] Thiele I , Sahoo S, Heinken A et al Personalized whole–body models integrate metabolism, physiology, and the gut microbiome. Mol Syst Biol 2020;16:e8982. 32463598 10.15252/msb.20198982 PMC7285886

[btae430-B39] Weitzel M , Nöh K, Dalman T et al 13CFLUX2 — high-performance software suite for ^13^C-metabolic flux analysis. Bioinformatics 2012;29:143–5. 23110970 10.1093/bioinformatics/bts646 PMC3530911

[btae430-B40] Wilkinson DJ. Bayesian methods in bioinformatics and computational systems biology. Brief Bioinf 2006;8:109–16. 10.1093/bib/bbm007 17430978

