# Supplementary material for: hopsy — a methods marketplace for convex polytope sampling in Python
Source: Bioinformatics. 2024 Jul 1;40(7):btae430. doi: 10.1093/bioinformatics/btae430 (PMC11245314; doi:10.1093/bioinformatics/btae430)
Supplement: btae430_Supplementary_Data [file btae430_supplementary_data.pdf]

Supplementary Information for

hopsy - a methods marketplace for convex polytope sampling in Python

Richard D. Paul [1,2], Johann F. Jadebeck [1,3], Anton Stratmann [1,3], Wolfgang Wiechert [1,3] and Katharina Nöh [1,*]

[1] Institute of Bio- and Geosciences, IBG-1: Biotechnology, Forschungszentrum Jülich, 52425 Jülich, Germany

[2] Institute of Advanced Simulations, IAS-8: Data Analytics and Machine Learning, Forschungszentrum Jülich, 52428 Jülich, Germany

[3] Computational Systems Biotechnology (AVT.CSB), RWTH Aachen University, 52062 Aachen, Germany

[*] Correspondence: k.noeh@fz-juelich.de

Contents

S.1 Code availability **2**

S.2 Constrained vs. Unconstrained Sampling **3**

S.3 Uniform Convex Polytope Sampling **5**
S.3.1 A Toy Example 5
S.3.2 Numerical Experiment 6
S.3.2.1 Results 6
S.3.3 Recon3D Benchmark 9

S.4 Bayesian ^{13}C-Metabolic Flux Analysis **10**
S.4.1 Numerical Experiment 10
S.4.1.1 Results 10

S.5 Composite Bioprocess and Metabolic Flux Modeling **14**
S.5.1 Numerical Experiment 15
S.5.1.1 Results 16

S.6Bibliography **17**

S.1 Code availability

The hopsy source code is available at https://jugit.fz-juelich.de/IBG-1/ModSim/hopsy under the MIT license with documentation https://modsim.github.io/hopsy/. Available sampling algorithms are continuously updated at https://modsim.github.io/hopsy/userguide/sampling.html\#proposals. The code for all example workflows described here is available at https://jugit.fz-juelich.de/IBG-1/ModSim/Fluxomics/hopsy-publication. This includes jupyter notebooks to reproduce all figures and all model files, accessible in the models directory. Further source code is available in the src subdirectory.

S.2 Constrained vs. Unconstrained Sampling

We emphasize the need for specialized convex polytope (CP) samplers by comparing the performance of unconstrained off-the-shelf samplers implemented in the popular Bayesian inference library `PyMC` against some of those provided in `hopsy`. For this, the density given by the negative exponential of the d-dimensional Rosenbrock function serves as test case [5]:

$$f(\theta) = \exp\left(-\sum_{i=1}^{d/2}[s \cdot (\theta_{2i-1}^2 - \theta_{2i})^2 + (\theta_{2i-1} - a)^2]\right) \tag{1}$$

with $s = 100$ and $a = 0$. For the constrained test-case, we limit θ to the unit cube $[0,1]^d$. In both cases, i.e., constrained and unconstrained, we test for $d \in \{2, 4, \ldots, 16, 18\}$.

Figure S.1 presents the performance comparison of the commonly used No-U-Turn sampler (NUTS) [9] with Gaussian proposal as implemented in `PyMC` [1], against the performance achieved with the Gaussian Hit-and-Run algorithm, designed for CP sampling, and the unconstrained Gaussian proposal implemented in `hopsy`. For both, we drew $10,000 \cdot d^2$ samples and ran 4 parallel Markov chains. The performance is measured in terms of relative effective sample size (ESS) per sample (n_{eff}/n), ESS per second (n_{eff}/s), and rank normalized split $\hat{R}$ [16]. Moreover, we report the achieved acceptance rate α.

For the unconstrained problem, we observe in Figure S.1 (left column) that our implementation of the unconstrained Gaussian proposal does not yield satisfying convergence ($\hat{R} > 2$ for 8 dimensions and higher), most likely due to a lack of step size tuning. `PyMC`, in contrast, performs some hyperparameter tuning, yielding better convergence for both variants tested.

For the constrained problem, we observe in Figure S.1 (right column) that the Gaussian sampler implemented in `PyMC` achieves the highest relative ESS per sample when the dimensionality is 8 or higher, but is superseded in terms of ESS per second by `hopsy`'s constrained Gaussian Hit-and-Run sampler in all tested dimensions. Importantly, with increasing dimension, the acceptance rates of `PyMC`'s Gaussian sampler progressively decline, which suggests that this sampler eventually will get stuck as the problem dimension further increases. Interestingly, the No-U-Turn sampler, while working well for unconstrained sampling, turns out to be a bad choice for constrained sampling, indicated by its high $\hat{R}$ statistics as well as very low ESS values. This is most likely due to the discontinuity along the CP borders.

Concluding, our numerical experiments with simple (orthogonal) constraints demonstrate the need for specialized constrained samplers, even in the case of relatively low problem dimensions.

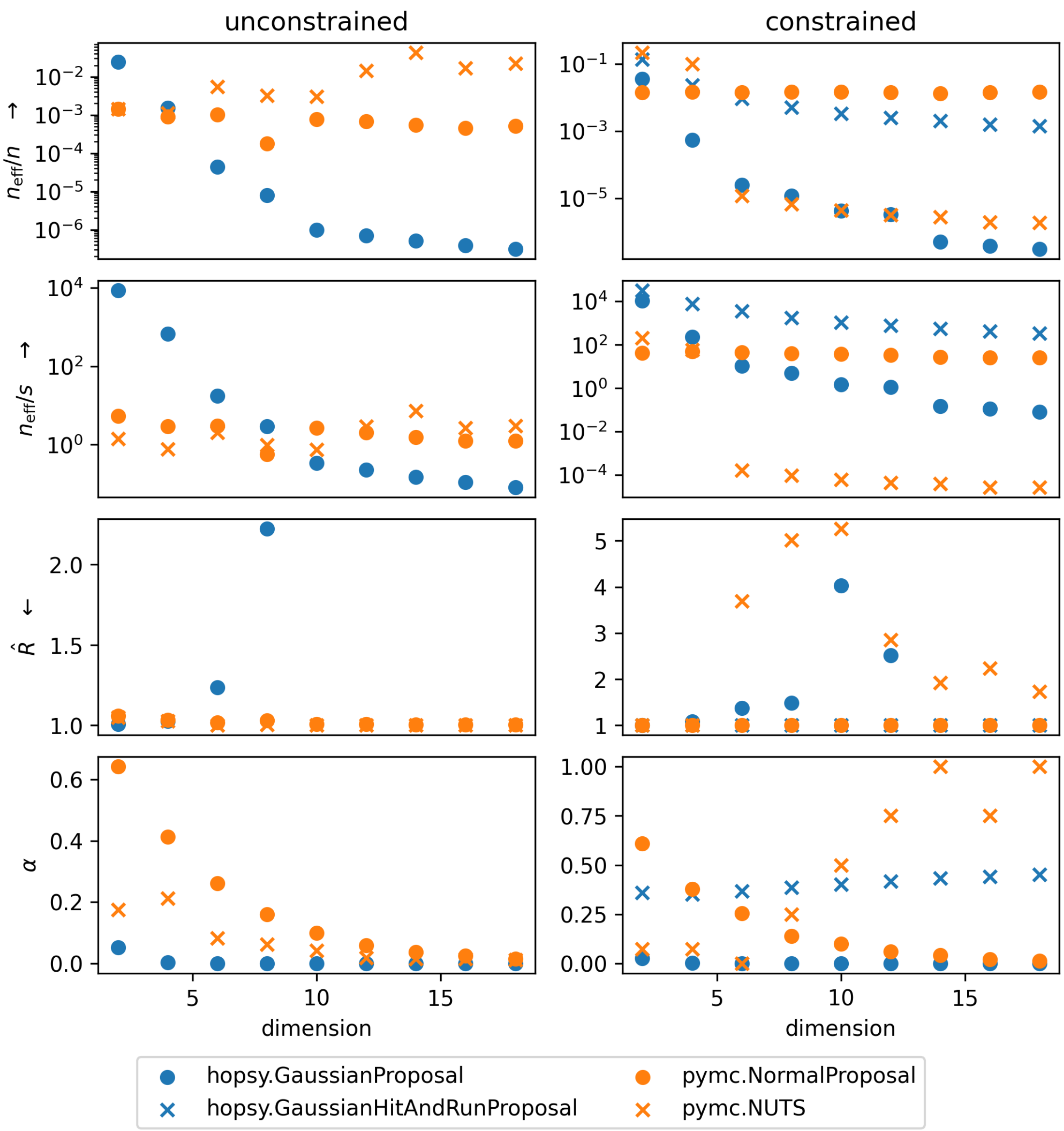

Figure S.1: Performance comparison of unconstrained off-the-shelf samplers implemented in `PyMC` against those implemented in `hopsy`.

S.3 Uniform Convex Polytope Sampling

As an example for deriving an explicit CP formulation from an implicit one, we consider the case of Metabolic Flux Analysis (MFA). MFA metabolic networks consist of a set of biochemical reactions. The evolution of intracellular metabolite concentrations X over time is described by a system of ordinary differential equations (ODE). These ODEs are derived from mass balancing of each reaction and parameterized by the metabolic reaction rates ν, which are also referred to as *fluxes*. The stoichiometric coefficients of the metabolites in each reaction are collected in the stoichiometric matrix S. Consequently, the ODE system is given as

$$\frac{\mathrm{d}X}{\mathrm{d}t} = S \cdot \nu \tag{2}$$

At metabolic steady state, the intracellular metabolic concentrations are considered constant. In this case, the dynamic system becomes an underdetermined linear equation system for the unknown fluxes ν:

$$0 = S \cdot \nu \tag{3}$$

The null space of S is parameterized by a subset of *free* fluxes θ, which are confined to a CP

$$\mathcal{P} = \{\theta : A \cdot \theta \leq b\} \tag{4}$$

Any $\theta \in \mathcal{P}$ is a feasible solution for Equation (3). Given no additional information, all solutions of Equation (3) are equally valid. In this case, from a probabilistic perspective, the feasible solutions are encoded by the uniform distribution over $\mathcal{P}$. In the case of Bayesian inference, the uniform distribution over $\mathcal{P}$ is a *proper* prior, if and only if the CP is bounded, which is generally ensured in practice. Within the specification of a `hopsy.Problem`, this corresponds to choosing a constant density $f(\theta) = \text{const}$. Notice that this case is implicitly assumed, if no `hopsy.Model` is passed to the `hopsy.Problem`.

S.3.1 A Toy Example

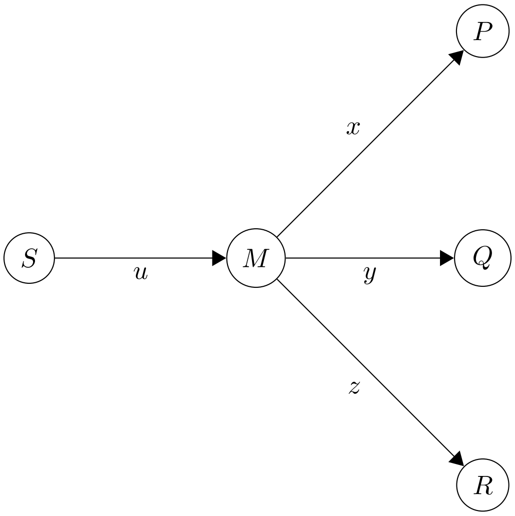

Figure S.2: A simple metabolic network. Upper-case letters denote metabolites and lower-case letters are metabolic reactions. Nominal reaction directions are indicated by the arrowhead. Here, the metabolite M is considered "intracellular" and therefore mass-balanced, with a constant concentration M, whereas the pools S, P, Q, and R are considered extracellular metabolites with time-dependent concentrations S, P, Q, R.

A toy example network is depicted in Figure S.2. Balancing the in- and outgoing fluxes of the internal metabolite M gives the stoichiometric matrix S

$$S = \begin{pmatrix} \overset{u}{1} & \overset{x}{-1} & \overset{y}{-1} & \overset{z}{-1} \end{pmatrix} M, \tag{5}$$

Normalizing the intracellular fluxes by the uptake rate u and assuming the fluxes x, y, z to go only in the nominal direction (arrow), we get

$$1 = \hat{x} + \hat{y} + \hat{z} \quad \text{and} \quad 0 \leq \hat{x}, \hat{y}, \hat{z} \leq 1 \tag{6}$$

as the solution of Equation (3) with $\hat{x} = x/u, \hat{y} = y/u, \hat{z} = z/u$. Selecting $\hat{x}$ and $\hat{y}$ as free fluxes, the space of feasible solutions is given by the inequality

$$1 \geq \hat{x} + \hat{y}, \tag{7}$$

which results in the CP $\mathcal{P}$ being a two-dimensional simplex

$$A \cdot \theta = \begin{pmatrix} 1 & 1 \\ -1 & 0 \\ 0 & -1 \end{pmatrix} \cdot \begin{pmatrix} \hat{x} \\ \hat{y} \end{pmatrix} \leq \begin{pmatrix} 1 \\ 0 \\ 0 \end{pmatrix} = b. \tag{8}$$

S.3.2 Numerical Experiment

We used the problem of feasible flux space sampling to highlight `hopsy`'s capabilities to rapidly prototype and implement MCMC algorithms in Python. In particular, we implemented a updated version of the little-considered Over-relaxed Hit-&-Run algorithm by [4] and a very recent iterative MCMC algorithm [3]:

- Over-relaxed Hit-&-Run with Rounding (ORHR),
- Multiphase Monte-Carlo algorithm using a Billiard walk proposal (MMBW)

We benchmarked both algorithms alongside `hopsy`'s native Coordinate Hit-&-Run with Rounding and Thinning algorithm (CHRRT) by uniformly sample three CP problems:

- a 16-dimensional Birkhoff polytope (`BP5`)
- a 18-dimensional metabolic flux model of *E. coli* from [20] (`zamboni`)
- a 24-dimensional constraint-based *E. coli* model from [13] (`e_coli_core`)

See `https://gitlab-public.fz-juelich.de/IBG-1/ModSim/Fluxomics/hopsy-publication/-/blob/main/Eval-use-case-1.ipynb` for definitions of the CPs and further details regarding this experiment.

All calculations were run on a system equipped with AMD 5950x CPU and running Ubuntu 22.04. For every of the three sampling algorithms, we ran four parallel chains, parallelized using Python's `multiprocessing` library. For the comparison of the three algorithms, each chain was run until an effective sample size (ESS) [6] of at least 1,000 was achieved. According to [10], the selection of the thinning factor impacts the algorithm performance considerably when rounding is involved, primarily due to the de-rounding step that maps the set of thinned samples back to the original parameter space. Here, following the guideline derived in [10], the thinning factor was set to $\mathit{problem\_dimension}^2 \times 1./6$ for CHRRT and ORHR. Because the MMBW algorithm rounding works differently, thinning was not applied in this case.

S.3.2.1 Results

Figure S.3 shows the relative ESS per sample (ESS/n) and per unit of time (in seconds, ESS/s) as measures of efficiency of three sampling algorithms used. Considering both measures, the Multiphase Billiard algorithm MMBW has the highest performance for `BP5` and `e_coli_core`. However, for the `zamboni` model, the CHRRT algorithm, which is built-in in `hopsy`, is most efficient. The marginal flux distributions of the three uniform sampling problems are shown in Figure S.4. In these plots the influence of the geometry of the CPs, on the marginal distributions becomes evident.

Both `e_coli_core` and `zamboni` are core models of *E. coli*, yet different algorithms perform best, showing that the performance of MCMC algorithms is problem specific. This motivates the development and inclusion of new algorithms in `hopsy`. Indeed, in view of the results in Figure S.3, the MMBW algorithm was natively included in `hopsy` version 1.4.0.

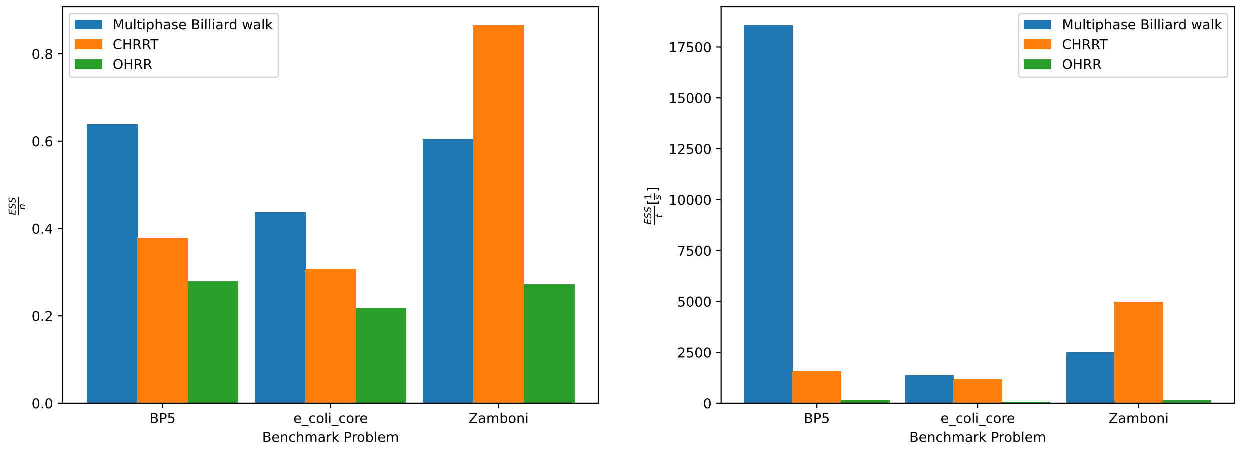

Figure S.3: Benchmark results. Relative effective sample size (ESS) per sample (left) and time measured in seconds (right) for the three algorithms (MMBW, CHRRT, ORHR) run on three problems (`BP5`, `e_coli_core`, `zamboni`).

(a) 16-dimensional Birkhoff polytope `BP5`.

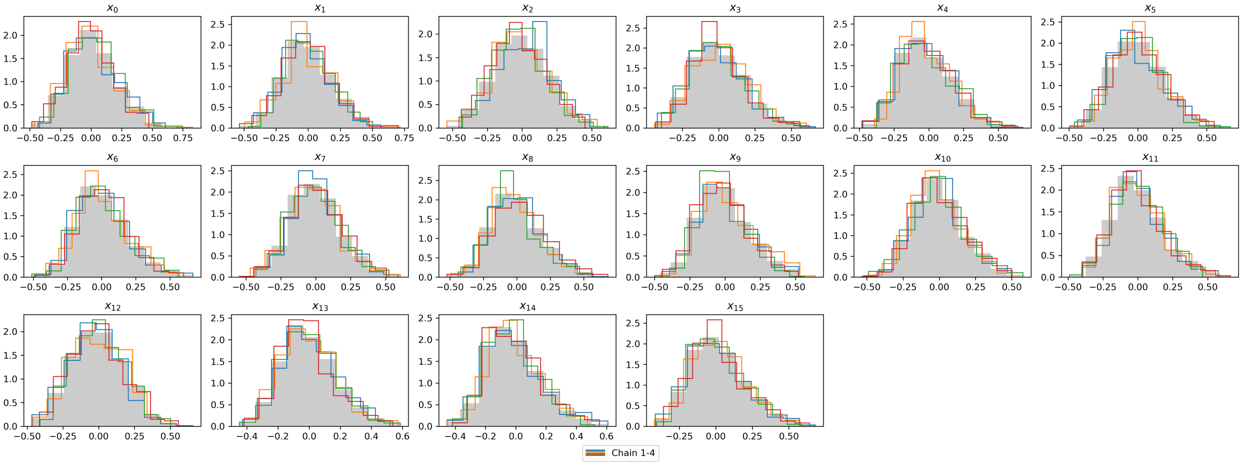

(b) *E. coli* model `zamboni`.

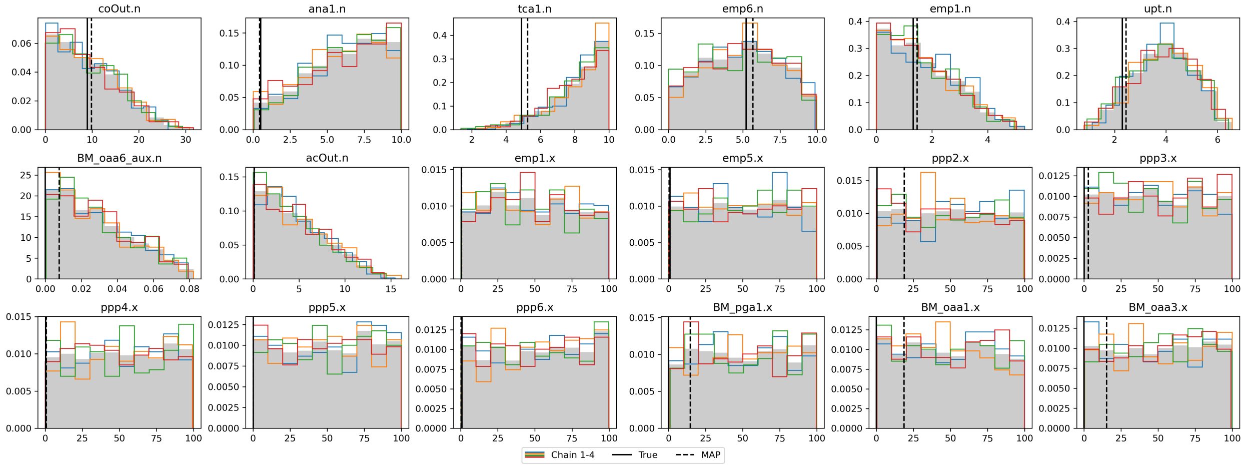

(c) *E. coli* model `e_coli_core`

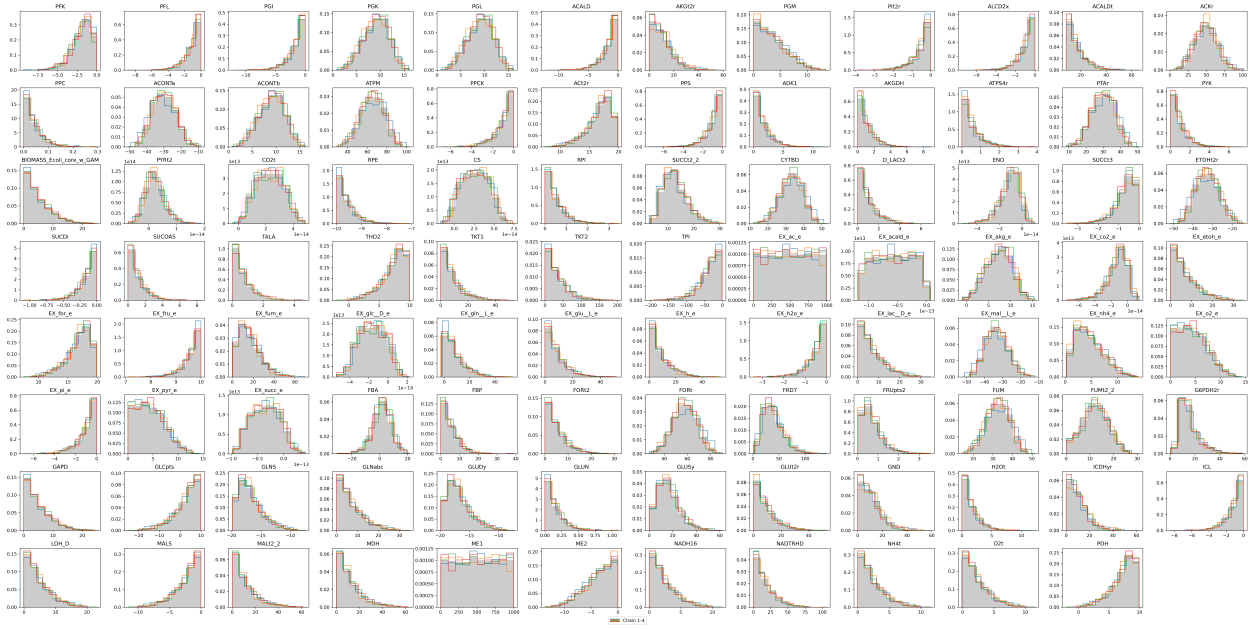

Figure S.4: Marginal distributions of random variables uniformly distributed on CP test problems. In (c) the full flux space, consisting of 95 fluxes, is shown.

S.3.3 Recon3D Benchmark

We used the CHRR algorithm [7] and the preprocessed `Recon3D` model files from [10] to benchmark `hopsy` for sampling large genome-scale models uniformly on a desktop PC (Intel i7-6700) and on the JURECA supercomputer at Jülich Supercomputing Centre (JSC). On the desktop PC, we used Python multiprocessing to run four independent chains in parallel on the four cores of the processor. On the supercomputer, we used MPI to parallelize the sampling on two so-called standard compute nodes, each equipped with 2 AMD EPYC 7742 processors, giving us 256 independent and parallel chains. Results are shown in Figure S.5.

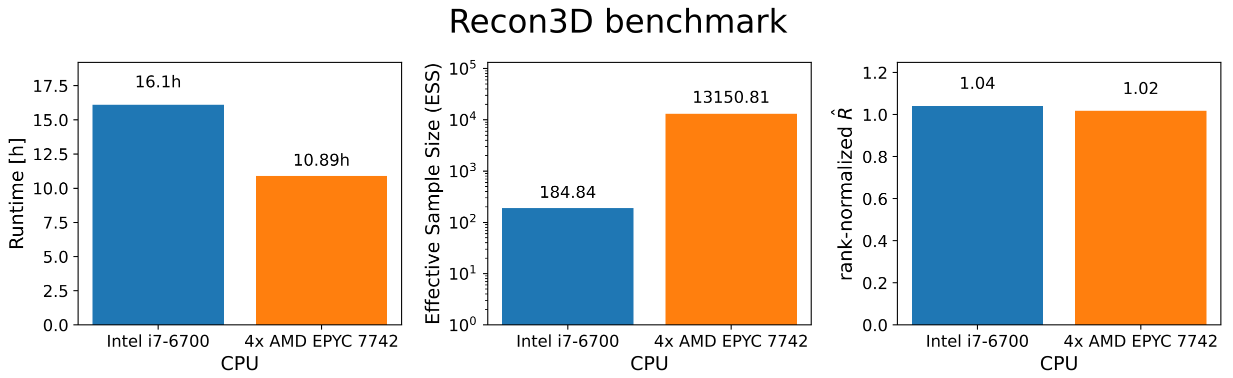

Figure S.5: Recon3D benchmark. Left: Run times for producing 40,000 samples per chain in the preprocessed space with a thinning constant of $10 * dim(Recon3D) = 10 * 4,861$. The back-transform to the full flux space was done by a one shot transformation, taking up only a fraction of the time (28 seconds for the desktop PC samples, and 235 seconds for the JURECA samples). In total, the desktop PC produced $40,000 * 4 = 360,000$ samples in 16 hours and the supercomputer produced $40,000 * 256 = 10,240,000$ samples in 11 hours. Middle: ESS obtained on the desktop PC and JURECA. A higher ESS is better. Because the chains are independent, the ESS scales with the number of chains. However, ESS estimates are only reliable for converged chains, which we checked by computing the rank-normalized split potential scale reduction factor $\hat{R}$ [16]. Right: $\hat{R}$ values are below 1.1 for the desktop PC run and JURECA, indicating acceptable mixing.

S.4 Bayesian ^{13}C-Metabolic Flux Analysis

Uniform flux space sampling, as described in Sec. S.3, represents the constraining impact of the model formulation on the fluxes, without considering measurements. In Bayesian ^{13}C-Metabolic Flux Analysis (MFA), two data types enter the inference problem. One data type is provided by the so-called *extracellular rates*. These rates represent the biomass-specific substrate uptake and product excretion rates, as well as the growth rate of the organism under study. The extracellular rates are extracted from exometabolomic concentration data, either by metabolite-wise linear regression [12] or by consistent inference using bioprocess models [8] (see also Sec. S.5).

While the knowledge of the extracellular rates is helpful, they are insufficient for determining the *intracellular fluxes* in parallel and cyclic metabolic pathways [19]. For this, the second data type is ^{13}C labeling data of intracellular metabolites that are acquired by administering carbon-based substrates in isotope labeling experiments. Specifically, ^{13}C-labeled substances are taken up by the cells, the label is propagated through the metabolic pathways according to the underlying fluxes, and chemical analytics (mass spectrometry or NMR) allows for the quantification of fractional enrichments of internal metabolites. On the other hand, the metabolic model is complemented with a carbon atom transition network, describing the fate of the individual carbon atoms through the metabolic network. This model is used to simulate the measurable isotope labeling states. Together, this allows estimating the fluxes by means of a computational procedure. Bayesian ^{13}C-MFA uses Bayes theorem to produce flux posterior probability distributions, see [15] for details.

The measurement errors on extracellular rate and labeling data are both modelled to be additive Gaussian. Formally, let Y_{13C} be the data vector of extracellular rate and isotope enrichment measurements, then

$$Y_{13C} \sim \mathcal{N}(g(\theta), \Sigma_{13C}) \tag{9}$$

where $g(\theta)$ is the vector of computed extracellular rates and simulated isotope labeling, given known fluxes θ, and Σ_{13C} is the associated diagonal measurement covariance matrix. Note, that the definition space of θ is restricted to a CP as detailed in Sec. S.3. Using the uniform distribution on the flux CP as prior, the log-density function for Bayesian ^{13}C-MFA (up to a constant) then is

$$\log f(\theta) = \frac{1}{2}\|Y_{13C} - g(\theta)\|^2_{\Sigma_{13C}} \tag{10}$$

Since simulation of isotope data by evaluating $g(\theta)$ is computationally expensive, we rely on the high-performance simulator 13CFLUX2 [18] for fast log-density evaluation.

S.4.1 Numerical Experiment

Using the `zamboni` model, we simulated isotope labeling data for an input tracer of [1,2]-^{13}C-labeled glucose. The reference fluxes and the measurement configuration were taken from [20] to simulate ground truth data, see model file at `https://jugit.fz-juelich.de/IBG-1/ModSim/Fluxomics/hopsy-publication/-/blob/main/models/Ecoli_NatProt_perturbed_small.fml?ref_type=heads`. The simulated rate and isotope labeling measurements were perturbed according to the Gaussian error model in Equation (9) using the standard deviation reported in [20].

We used an Intel(R) Xeon(R) CPU E5-2683 v4 @ 2.10GHz running Ubuntu 22.04 to simulate 64 chains in parallel twice. In the first (non-PT) run, all chains were independent. In the second (PT) run, we let the chains communicate by binding the chains together in sets of 16. In both experiments, we sampled 1e6 samples with a thinning of 20 using the Hit-and-Run with Rounding algorithm and discarded the first half of the chains as burn-in.

S.4.1.1 Results

Both MCMC runs, PT and non-PT, converged, as indicated by the potential scale reduction factor $\hat{R}$ [6], and the trace plots in Figure S.6. For the independent chains, the $\hat{R}$ was below 1.02 after 10.8 h, indicating convergence. The PT run achieved an $\hat{R}$ below 1.002 in 12.8 h, therefore mixed better for the same number of samples drawn with around 1.2 times longer run-time.

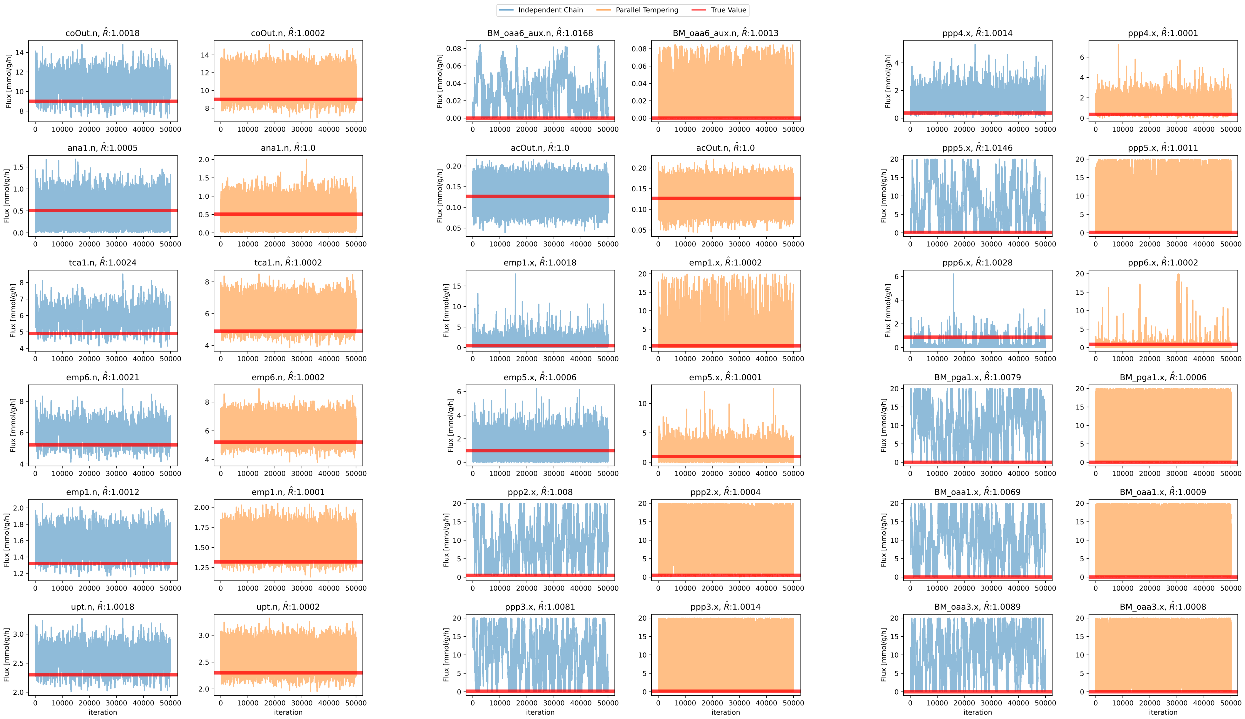

Figure S.6: Trace plots of MCMC simulations without (blue) and with parallel tempering (orange). The horizontal red line represents the reference fluxes. High confidence in MCMC convergence is visible as a 'lack of structure" in the trace plots, i.e. little auto-correlation of the fluxes. For some fluxes, such as `ppp2.x`, the reduction in auto-correlation from using parallel tempering is clearly visible. For each flux, the rank normalized potential split scale reduction factors $\hat{R}$ were computed using **`Arviz`** [11].

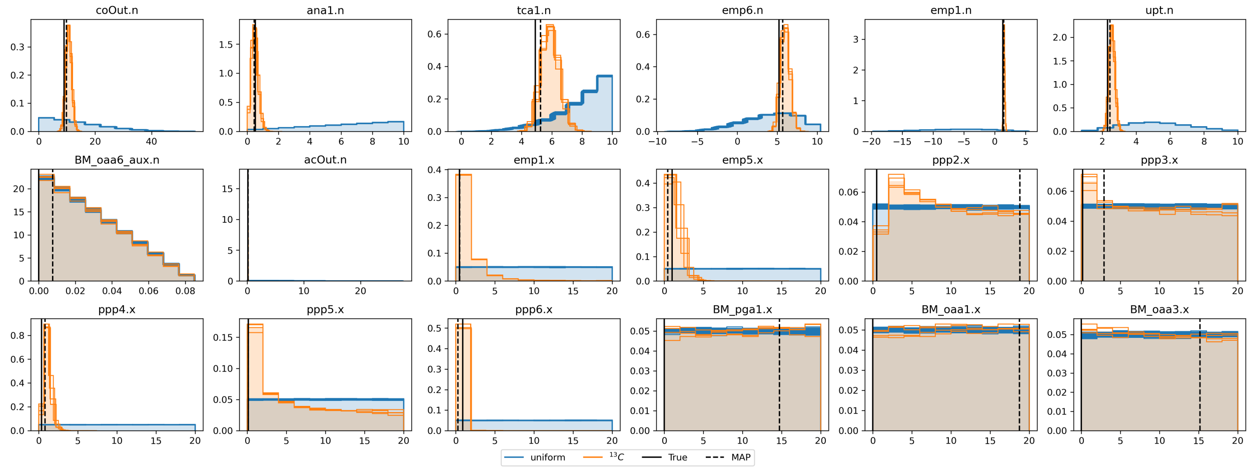

Figure S.7: Comparison of marginal distributions for the independent fluxes of the `zamboni` model. Uniform (prior) marginal distributions (blue) and posterior marginal distributions (orange). The reference fluxes are indicated by a vertical dashed line. The comparison emphasizes the information gain in major fluxes (primarily net fluxes), obtained from taking ^{13}C-MFA data into account.

The marginal distributions obtained from sampling the ^{13}C-MFA posterior (Equation (10)) are shown in Figure S.7. To study the information gain obtained from taking extracellular rates and isotope labeling data into account, we compare the marginal flux posterior distributions with the marginal flux prior distributions (cf. Section S.3). Except for six of the 18 fluxes, one net flux (`BM_oaa6_aux.n`) and five exchange fluxes (`ppp2.x`, `ppp3.x`, `BM_pga1.x`, `BM_oaa1.x`, `BM_oaa3.x`), a clear reduction in variance is observable. Notice that exchange fluxes are indeed notoriously hard to identify [19].

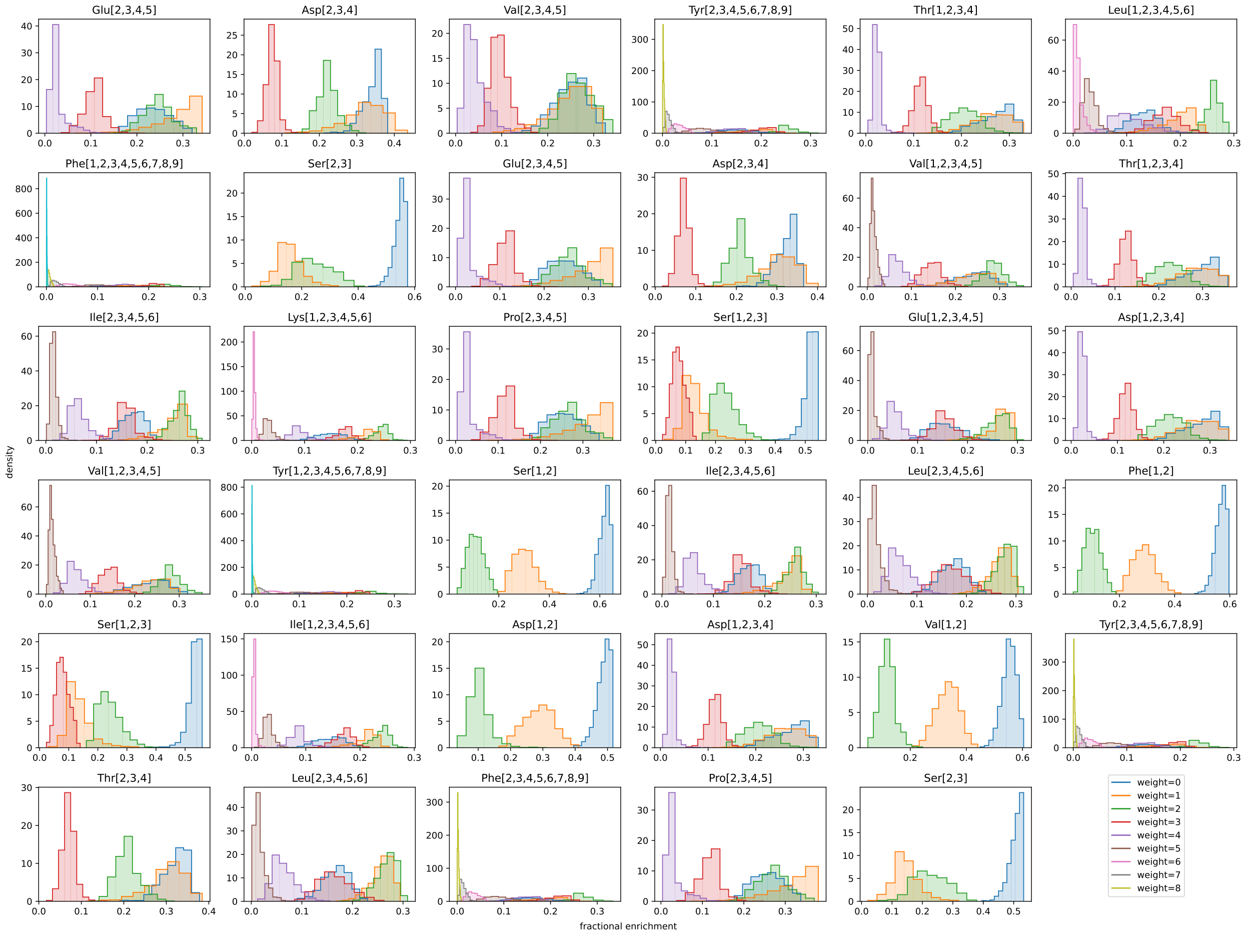

Figure S.8: Sample-based prediction of measurements as part of experimental design.

Finally, we show how sampling results contribute to experimental design decisions. As an example, Figure S.8 shows the isotope labeling data predicted for [1,2]-^{13}C-labeled glucose as tracer, by re-using the results generated by uniform sampling before (cf. Sec. S.3). In the same way, arbitrary tracer mixture can be tested, predicting likely isotope labeling patterns to emerge, being a valuable information source for analytical experts [14].

S.5 Composite Bioprocess and Metabolic Flux Modeling

In ^{13}C-MFA, intracellular fluxes are traditionally determined sequentially: first, extracellular rates are estimated using linear regression or a bioprocess model, then, the inferred rates along with isotope labeling data are used for estimating the intracellular flux using a ^{13}C-MFA model (cf. Section S.4). This sequential process, however, neglects the correlation between those parameters that are shared between the bioprocess and the ^{13}C-MFA models, i.e. the extracellular rates. In turn, the neglection might lead to an information bias. To improve the reliability of the parameter estimates, we here, for the first time, simultaneously inferred bioprocess and ^{13}C-MFA parameters, exploiting `hopsy`'s trampoline mechanism. Finally, we compared the inferences using the composite model with those of the sequential approach.

A bioprocess model is a kinetic model that balances the dynamic substance concentrations X in a bioreactor, starting from initial concentrations X_0:

$$\frac{\mathrm{d}X}{\mathrm{d}t} = h(X, \theta_{bp}), \quad X(0) = X_0 \tag{11}$$

where h is a nonlinear function of kinetic parameters θ_{bp}, which describes the time-dependent "mechanistics" of biomass and extracellular compound concentrations in a cultivation, such as exponential biomass growth or Monod substrate uptake kinetics. Given a time-series of extracellular biomass and concentration measurements Y_{bp} with additive Gaussian errors, we have

$$Y_{bp} \sim \mathcal{N}(X(\theta_{bp}), \Sigma_{bp}) \tag{12}$$

with $X(\theta_{bp})$ being the solution of Equation (11) for given kinetic parameters θ_{bp} and the (diagonal) measurement covariance matrix Σ_{bp}.

In contrast to bioprocess models that balance time-dependent extracellular concentrations within a bioreactor, ^{13}C-MFA models balance the metabolite concentrations within a cell that is in a metabolic (pseudo)steady state (cf. Section S.4). The precondition to connect bioprocess and ^{13}C-MFA models is, thus, that in the time interval in which isotope labeling is administered, the rate of change in extracellular concentrations $h(X, \theta_{bp})$ is approximately constant, suggesting that the cells' uptake, secretion, and growth rates remain reasonably constant. We check this criterion by computing the maximal deviation from the mean rates during the labeling interval. If the maximal deviation does not exceed a tolerable threshold, we expect that the bioprocess shows constant growth, and thus, the cells are in a metabolic steady state.

If this precondition is fulfilled, ^{13}C-MFA (Equation (9)) and bioprocess (Equation (12)) measurements are combined to the joint measurement vector Y and the joint measurement covariance matrix, resulting in the composite statistical model

$$Y = \begin{pmatrix} Y_{13C} \\ Y_{bp} \end{pmatrix} \sim \mathcal{N}\left(\begin{pmatrix} g(\theta_{13C}) \\ X(\theta_{bp}) \end{pmatrix}, \begin{pmatrix} \Sigma_{13C} & 0 \\ 0 & \Sigma_{bp} \end{pmatrix} \right) \tag{13}$$

With the joint parameter vector θ now constituted by the parameters of the bioprocess and ^{13}C-MFA models, this gives the composite posterior log density

$$\log f(\theta) = \begin{cases} \frac{1}{2}\|Y_{bp} - X(\theta_{bp})\|^2_{\Sigma_{bp}} + \frac{1}{2}\|Y_{13C} - g(\theta_{13C})\|^2_{\Sigma_{13C}} & \text{if } h(X, \theta_{bp}) \approx \text{const. and } \theta_{13C} \in \mathcal{P}, \\ -\infty & \text{else,} \end{cases} \tag{14}$$

where $g(\theta_{13C})$ is the forward simulation of rate and labeling measurements of an isotope labeling experiment with the ^{13}C-MFA model, as described in Section S.4.

Note, that the composite model introduces an implicit dependency between the parameters of the ^{13}C-MFA model and the parameters of the bioprocess model, namely $\theta_{13C} = \theta_{13C}(\theta_{bp})$. That is, extracellular fluxes are computed from the bioprocess parameters, whereas intracellular fluxes remain free parameters that are to be inferred directly. We make this more explicit for actual models in Equation (18). Here, θ_{13C} has to satisfy the linear constraints introduced in Section S.3. Since the dependency is nonlinear in θ_{bp}, the condition $\theta_{13C} \in \mathcal{P}$ has to be tested for every new parameter estimate θ_{bp}.

S.5.1 Numerical Experiment

We combined the previously used `zamboni` model of *E. coli* with a chemostat bioprocess model, which is given by the following system of ordinary differential equations (adapted from [2]),

$$\frac{\mathrm{d}}{\mathrm{d}t}\begin{pmatrix} S \\ X \\ P \\ Q \end{pmatrix} = \begin{pmatrix} -\left(\frac{\mu}{Y_{XS}} + \frac{q_p}{Y_{PS}} + \frac{q_q}{Y_{QS}}\right) \cdot X + (S_f - S) \cdot D, \\ q_p \cdot X - D \cdot P, \\ (\mu - D) \cdot X, \\ q_q \cdot X - D \cdot Q, \end{pmatrix} \tag{15}$$

where S is the substrate (glucose), X the biomass, and P (acetate) as well as Q (CO_2) the extracellular product concentrations. Initial concentrations are $S(0) = 5000$ [mM], $X(0) = 2.5$ [g/L], $P(0) = 0.0$ [mM], and $Q(0) = 0.0$ [mM]. The substrate feed was fixed to $S_f = 10$ [mM] and the dilution rate was set equal to the growth rate, $D = \mu = 0.32$ [1/h].

Hence, the parameters θ_{bp} of the bioprocess model are the growth rate μ [1/h], yield coefficients Y_{XS} [gCDW/mmol], Y_{PS} [gCDW/mmol], and Y_{QS} [gCDW/mmol], as well as product formation rates q_p [mmol/gCDW/h] and q_q [mmol/gCDW/h]:

$$\theta_{bp} = (\mu, Y_{XS}, Y_{PS}, Y_{QS}, q_q, q_p). \tag{16}$$

On the other hand, the ^{13}C-MFA model is parameterized with the same free fluxes as selected before in Section S.4, namely

$$\begin{aligned} \theta_{13C} = (\quad & upt.n, emp1.n, emp6.n, tca1.n, ana1.n, acOut.n, coOut.n, BM\_oaa6\_aux.n, \\ & emp1.x, emp5.x, ppp2.x, ppp3.x, ppp4.x, ppp5.x, ppp6.x, \\ & BM\_pga1.x, BM\_oaa1.x, BM\_oaa3.x) \end{aligned} \tag{17}$$

To combine the bioprocess and the ^{13}C-MFA models, we associate extracellular substrate decline and product formation rates with the specific uptake rate, *upt.n*, and the two specific secretion net fluxes, *acOut.n* and *coOut.n*, of the ^{13}C-MFA model. That is, we map

$$\begin{aligned} upt.n(\theta_{bp}) &= \tfrac{\mu}{Y_{XS}} + \tfrac{q_p}{Y_{PS}} + \tfrac{q_q}{Y_{QS}} \\ acOut.n(\theta_{bp}) &= q_p \\ coOut.n(\theta_{bp}) &= q_q, \end{aligned} \tag{18}$$

yielding the joint parameter vector

$$\begin{aligned} \theta = (\quad & \mu, Y_{CS}, Y_{PS}, Y_{QS}, q_q, q_p, \\ & emp1.n, emp6.n, tca1.n, ana1.n, BM\_oaa6\_aux.n, \\ & emp1.x, emp5.x, ppp2.x, ppp3.x, ppp4.x, ppp5.x, ppp6.x, \\ & BM\_pga1.x, BM\_oaa1.x, BM\_oaa3.x) \end{aligned} \tag{19}$$

As the rates of the chemostat model are constant with respect to the concentration changes, constancy in growth conditions is ascertained, rendering the test in (14) naturally fulfilled.

Labeling measurements and extracellular rate measurements for the ^{13}C-MFA model were simulated as described before in Section S.4. Bioprocess concentrations were simulated with parameters θ_{bp} that reproduces the previous ^{13}C-MFA parameters using Equation (18). The simulated values were then perturbed with normally distributed $\epsilon \sim \mathcal{N}(0, \sigma)$, where σ was set to 0.1 for X, P and Q, 0.02 for S, and the ^{13}C-MFA measurements as described in Section S.4. All bioprocess parameters were bounded within a range of $[0, 20]$. ^{13}C-MFA model bounds were as before in Section S.3 and Section S.4. The bioprocess model in Equation (11) is solved by a `SciPy` [17] integrator, the `zamboni` model is simulated with 13CFLUX2. Glue code to implement the composite model was written in Python and can be found at `https://jugit.fz-juelich.de/IBG-1/ModSim/Fluxomics/hopsy-publication/-/blob/main/src/bioprocciso_pt.py?ref_type=heads`.

We used an Intel(R) Xeon(R) Gold 6130 CPU @ 2.10GHz running Ubuntu 22.04 to simulate 64 chains in parallel. Because parallel tempering performed well for inference of the ^{13}C-MFA model, we also use parallel tempering and let chains communicate in sets of 16, obtaining four replicates. We generated 1e6 samples with a thinning of 20 using the Hit-and-Run algorithm and discarded the first half of the chains as burn-in. The run converged with a potential scale reduction factor ($\hat{R}$) below 1.08 in around 55 hours. Since our case study was focused on rapid prototyping, less emphasis was laid on run time performance. Clearly, the run time can be improved by optimizing the simulation code.

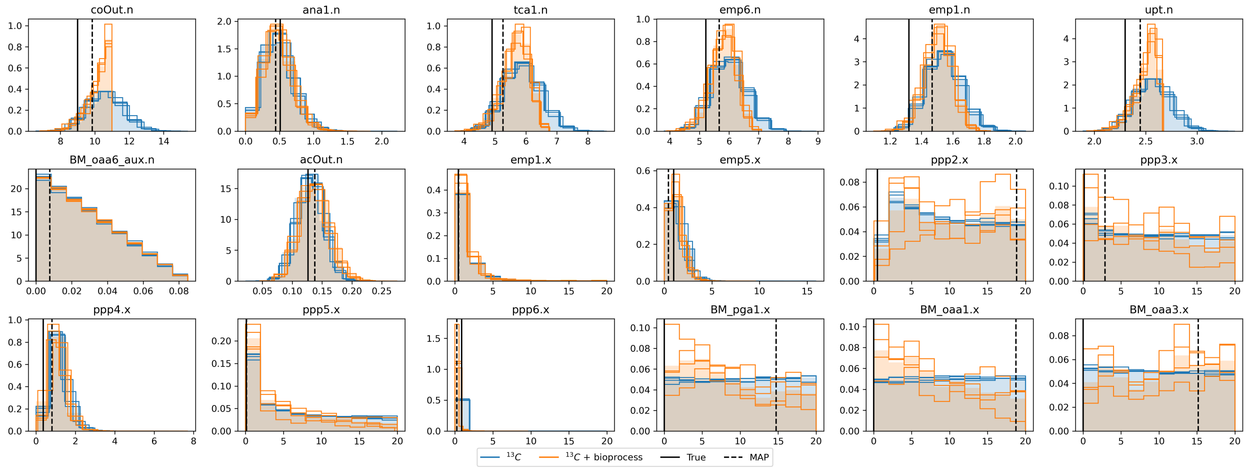

Figure S.9: Comparison of posterior marginal distributions of fluxes of the `zamboni` ^{13}C-MFA model obtained using the sequential approach, described in Section S.4, versus marginal distributions obtained from the composite modeling approach (cf. Section S.5). The black solid line indicates the true value used to generate the simulated data, the black dashed line indicates the *maximum a posteriori* (MAP) estimate of the composite model. The comparison emphasizes additional information gain in major fluxes (primarily net fluxes), obtained from taking the coupling of bioprocess and ^{13}C-MFA modeling into account.

S.5.1.1 Results

In Figure S.9,marginal distributions of fluxes obtained from sampling Equation (14) are shown. Note, that the fluxes *upt.n*, *acOut.n* and *coOut.n* are functions of the sampled bioprocess parameters θ_{bp} (cf. Equation (18)). Thus, for these fluxes we depict the marginal forward distributions obtained from applying the map Equation (18) to the posterior samples of the bioprocess parameters. Notably, we observe slightly improved identifiability for some fluxes, in particular the rates *coOut.n* and *upt.n*, but also the intracellular fluxes *tca1.n*, *emp6.n* and *emp1.n*. These results suggest that combining models as proposed here can improve flux estimations.

S.6 Bibliography

[1] O. Abril-Pla, V. Andreani, C. Carroll, L. Dong, C.J. Fonnesbeck, M. Kochurov, R. Kumar, J. Lao, C.C. Luhmann, O.A. Martin, M. Osthege, R. Vieira, T. Wiecki, and R. Zinkov. PyMC: A modern and comprehensive probabilistic programming framework in Python. *PeerJ Computer Science*, 9:e1516, 2023.

[2] A. Ajbar and A. Fakeeha. Static and dynamic behavior of a class of unstructured models of continuous bioreactors with growth associated product. *Bioprocess and Biosystems Engineering*, 25(1):21–27, 2002.

[3] A. Chalkis and V. Fisikopoulos. VolEsti: Volume approximation and sampling for convex polytopes in R. *The R Journal*, 13(2):642–660, 2021.

[4] G. de Concini and D. de Martino. Over-relaxed Hit-and-Run Monte Carlo for the uniform sampling of convex bodies with applications in metabolic network biophysics. *International Journal of Modern Physics C*, 26(1):1550010, 2015.

[5] L. C. W. Dixon and D. J. Mills. Effect of rounding errors on the variable metric method. *Journal of Optimization Theory and Applications*, 80(1):175–179, January 1994.

[6] A. Gelman. *Bayesian Data Analysis*. CRC Press, Boca Raton, 2014.

[7] H.S. Haraldsdóttir, B. Cousins, I. Thiele, R.M.T. Fleming, and S. Vempala. CHRR: Coordinate Hit-and-Run with rounding for uniform sampling of constraint-based models. *Bioinformatics*, 33(11):1741–1743, 2017.

[8] J. Hemmerich, N. Tenhaef, W. Wiechert, and S. Noack. pyFOOMB: Python framework for object oriented modeling of bioprocesses. *Engineering in Life Sciences*, 21(3-4):242–257, 2021.

[9] M.D. Hoffman and A. Gelman. The No-U-Turn sampler: Adaptively setting path lengths in Hamiltonian Monte Carlo. *Journal of Machine Learning Research*, 15(1):1593–1623, 2014.

[10] J.F. Jadebeck, W. Wiechert, and K. Nöh. Practical sampling of constraint-based models: Optimized thinning boosts CHRR performance. *PLOS Computational Biology*, 19(8):e1011378, 2023.

[11] R. Kumar, C. Carroll, A. Hartikainen, and O. Martin. Arviz a unified library for exploratory analysis of Bayesian models in Python. *Journal of Open Source Software*, 4(33):1143, 2019.

[12] T.A. Murphy and J.D. Young. ETA: Robust software for determination of cell specific rates from extracellular time courses. *Biotechnology and Bioengineering*, 110(6):1748–1758, 2013.

[13] Jeffrey D. Orth et al. Reconstruction and use of microbial metabolic networks: the core *Escherichia coli* metabolic model as an educational guide. *EcoSal Plus*, 4(1), 2010.

[14] J. Schellenberger, D.C. Zielinski, W. Choi, S. Madireddi, V. Portnoy, D.A. Scott, J.L. Reed, A.L. Osterman, and B.Ø Palsson. Predicting outcomes of steady-state ^{13}C isotope tracing experiments using Monte Carlo sampling. *BMC Systems Biology*, 6(1):9, Jan 2012.

[15] A. Theorell, S. Leweke, W. Wiechert, and K. Nöh. To be certain about the uncertainty: Bayesian statistics for ^{13}C metabolic flux analysis. *Biotechnology and Bioengineering*, 114(11):2668–2684, 2017.

[16] A. Vehtari, A. Gelman, D. Simpson, B. Carpenter, and P.-C. Bürkner. Rank-normalization, folding, and localization: An improved $\widehat{R}$ for assessing convergence of mcmc. *Bayesian Analysis*, 2020.

[17] P. Virtanen, R. Gommers, T.E. Oliphant, M. Haberland, T. Reddy, D. Cournapeau, E. Burovski, P. Peterson, W. Weckesser, J. Bright, S.J. van der Walt, M. Brett, J. Wilson, K.J. Millman, N. Mayorov, A.R.J. Nelson, E. Jones, R. Kern, E. Larson, C.J. Carey, İ. Polat, Y. Feng, E.W. Moore, J. VanderPlas, D. Laxalde, J. Perktold, R. Cimrman, I. Henriksen, E.A. Quintero, C.R. Harris, A.M. Archibald, A.H. Ribeiro, F. Pedregosa, P. van Mulbregt, and SciPy 1.0 Contributors. SciPy 1.0: Fundamental algorithms for scientific computing in Python. *Nature Methods*, 17:261–272, 2020.

[18] M. Weitzel, K. Nöh, T. Dalman, S. Niedenführ, B. Stute, and W. Wiechert. 13CFLUX2 — high-performance software suite for ^{13}C-metabolic flux analysis. *Bioinformatics*, 29(1):143–145, 2012.

[19] W. Wiechert, S. Niedenführ, and K. Nöh. A primer to ^{13}C metabolic flux analysis. In *Fundamental Bioengineering*, pages 97–142. Wiley-VCH Verlag GmbH & Co. KGaA, 2015.

[20] N. Zamboni, S.-M. Fendt, M. Rühl, and U. Sauer. ^{13}C-based metabolic flux analysis. *Nature Protocols*, 4(6):878–892, 2009.
